# A Palladium-Tin Modified Microband Electrode Array for Nitrate Determination

**DOI:** 10.3390/s150923249

**Published:** 2015-09-15

**Authors:** Yexiang Fu, Chao Bian, Jian Kuang, Jinfen Wang, Jianhua Tong, Shanhong Xia

**Affiliations:** 1 State Key Laboratory of Transducer Technology, Institute of Electronics, Chinese Academy of Sciences, Beijing 100190, China; E-Mails: akechi89@163.com (Y.F.); renzhongxing1@163.com (J.K.); binfen328@163.com (J.W.); jhtong@mail.ie.ac.cn (J.T.); shxia@mail.ie.ac.cn (S.X.); 2 University of Chinese Academy of Sciences, Beijing 100080, China

**Keywords:** nitrate determination, bimetallic composite, palladium-tin coexistence, electrodeposition, microband electrode array

## Abstract

A microband electrode array modified with palladium-tin bimetallic composite has been developed for nitrate determination. The microband electrode array was fabricated by Micro Electro-Mechanical System (MEMS) technique. Palladium and tin were electrodeposited successively on the electrode, forming a double-layer structure. The effect of the Pd-Sn composite was investigated and its enhancement of catalytic activity and lifetime was revealed. The Pd-Sn modified electrode showed good linearity (R^2^ = 0.998) from 1 mg/L to 20 mg/L for nitrate determination with a sensitivity of 398 μA/(mg∙L^−1^∙cm^2^). The electrode exhibited a satisfying analytical performance after 60 days of storage, indicating a long lifetime. Good repeatability was also displayed by the Pd-Sn modified electrodes. The results provided an option for nitrate determination in water.

## 1. Introduction

Fresh water is of great importance to people’s life and production. With the development of industry and agriculture, water contamination has become more and more serious. Nitrate is one of the common contaminants in water because of its high solubility. Accumulation of nitrate in water may lead to eutrophication [1] or even algal bloom and its reduction product of nitrite may lead to serious diseases to the human body including liver diseases, cancer and blue baby syndrome [2]. Therefore, there are growing concerns on nitrate determination and nitrate removal in water resources. The world health organization has recommended that the nitrate level in potable water should not exceed 11 mg·L^−1^ (as N). The US environmental protection agency has set a limit of 10 mg·L^−1^ (as N) for nitrate concentration in drinking water [3].

There are many methods for the determination of nitrate and most of them are based on techniques such as spectrophotometry, electrochemistry and chromatography [4]. The electrochemical method stands outs by its simple operation, low price and speediness. To increase the sensitivity of the electrode for nitrate determination, modification on the electrode is required. Metal materials such as copper [5,6,7] and silver [8,9,10] are the major modification materials modified on the electrodes. Copper is inexpensive and has been widely investigated. It shows relatively high sensitivity and good linearity. Silver is also effective for nitrate determination and could be used in neutral and alkaline medium. Nevertheless, this kind of electrode suffers from poor stability and short lifetime, as fresh copper and silver surfaces are more effective for cathodic reactions owing to easy oxidation of the modification layer [11,12]. For example, Guadagnini *et al.* deposited silver nanoparticles (AgNPs) on graphite sheets and bulk glass carbon electrodes for nitrate determination [9], the lifetime of which was considered only one day.

To overcome the problem of lifetime, researchers have found sophisticated ways to renew copper catalysts of the electrode. A fresh modification layer was electrodeposited on the electrode prior to each analysis by introducing solution with copper ions into the sample, which would lead to sample contamination [13]. Some researchers used a plating solution of defined composition to *ex situ* regenerate the modification layer on the electrode surface regularly [14]. However, these renewing procedures make nitrate sensing more complex. A kind of catalyst that keeps stable after long-time storage is necessary for the long lifetime of the nitrate sensor.

Bimetallic composite has been investigated for the removal of nitrate in water because of its effectively catalytic activity [15,16,17,18,19,20,21,22,23,24]. The bimetallic composite generally consists of a noble metal and a promoting transition metal. The role of the transition metal is to reduce nitrate according to a redox process leading to its oxidation; the noble metal helps to stabilize the transition metal in its reduction stage for its ability to absorb hydrogen [15]. Bimetallic composites, including PdCu [16], PtRh [17], PtAg [18] *etc.* generally exhibit higher catalytic activity than monometal catalysts such as Cu [5], Ag [8], Sn [25]. Particularly, Pd-M (M refers to Cu, Sn, Ge) shows higher catalyst activity [19]. Pd-Sn bimetallic composite has been investigated for nitrate removal [20,21,22,23,24] and showed an extremely high electrocatalytic activity for the reduction of nitrate.

In this paper, Pd-Sn bimetallic composite was chosen as the modification material for nitrate determination. The purpose of this work is to improve the sensitivity and the stability of the electrode by developing Pd-Sn bimetallic composite. As indicated above, palladium could help to stabilize the sensing activity of tin for nitrate reduction. Tin is also favored for its properties such as non-toxicity, high corrosion resistance [26] and stability [20] exposed in air and in water. These properties could make it possible that the sensing electrode modified with Pd-Sn bimetallic composite keeps effective after long-time storage.

Microelectrode has its properties and advantages such as fast response, low background noise and high current density [27,28]. Microelectrode array with the advantages of microelectrode is developed to obtain a higher current output and it has been used for numerous applications including biochemical and environmental analysis [29]. In the previous work of our lab, microband electrode arrays modified with silver [30] and copper [31] were used for nitrate determination. Their research results proved that microband electrodes were beneficial for the sensitivity of the nitrate sensor.

In this work, microband electrode arrays were used as the basic electrode of the working electrode for nitrate determination. Palladium-tin bimetallic composite was electrodeposited on the microelectrode as the sensing material. The modified electrode was investigated for its linearity for nitrate determination. Some other properties of the nitrate sensor, repeatability and lifetime included, were also investigated.

## 2. Experimental Section

### 2.1. Materials and Chemicals

All solid reagents were of analytical grade and were used without further purification. Ethanol, chlorhydric acid (36%~38%) and sulfate acid were obtained from Beijing Chemical Works; SnCl_2_, NaCl, HClO_4_, KNO_3_ and tin granular were obtained from Sinopharm Chemical Reagent Co. Ltd., Shanghai, China; PdCl_2_ was obtained from Sigma-Aldrich; water used in the experiment was all deionised water with a resistivity of 18 MΩ·cm obtained from Millipore Direct-Q 3 UV system.

### 2.2. Apparatus

A three-electrode system based on microband electrode arrays was used for the electrochemical experiments. The working electrode is the microband electrode array fabricated by MEMS technique in our lab. Platinum was deposited on glass substrate and then patterned by lift-off process to form the microband array structure. The length of the microband was 2000 μm and the width was 10 μm. The gap between the bands was 50 μm. The total number of the bands was 50. The working electrode was defined by a layer of SU-8 negative photoresist, thus exhibiting a fixed effective area of 1 mm^2^. The counter electrode was a PTFE-shrouded platinum disk electrode with a diameter of 3 mm. A KCl saturated Ag/AgCl reference electrode acted as a reference, and all potentials reported are referred to here. All electrochemical experiments were controlled by electrochemical measurement system CHI620e. The data were analyzed using CHI620e electrochemical analyzer software. A pHS-3C meter with digital display was used to determine the pH of the electrolyte. An optical microscope was used to preliminarily observe the surface of the electrode. Scanning electron microscopy (SEM) and energy dispersive spectroscopy (EDS) analysis of the deposited layer was performed using an S-4800 field emission scanning electron microscope (FESEM) produced by Hitachi (Tokyo, Japan).

### 2.3. Working Electrode Modification

The fabricated microband electrode array was electrochemically cleaned in 0.05 M H_2_SO_4_ by cycling the electrode potential between −0.4 V and 1.2 V at a scan rate of 50 mV/s until a stable cyclic voltammogram was obtained. Then palladium was electrodeposited on the working electrode by applying a fixed potential of −0.56 V for 110 s. The electrolyte was 20 mM PdCl_2_ dissolved in 1 M NaCl. After the palladium modified electrode was rinsed with deionised water, tin was subsequently deposited on palladium layer by cyclic voltammetry for 20 cycles in aqueous solution containing 20 mM SnCl_2_ and 0.1 M HCl, with a potential ranging from −0.45 V to −0.58 V and a scan rate of 50 mV/s. In the same electrolyte, a tin modified electrode was prepared by cyclic voltammetry from −0.45 V to −0.58 V for 20 cycles on a bare Pt microband electrode array. This electrode served as a contrast.

## 3. Results and Discussion

### 3.1. Preparation of Pd-Sn Electrode

#### 3.1.1. Electrodeposition of Palladium

**Figure 1:**
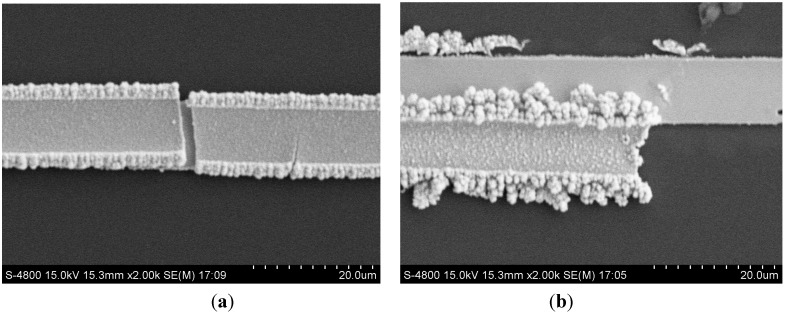

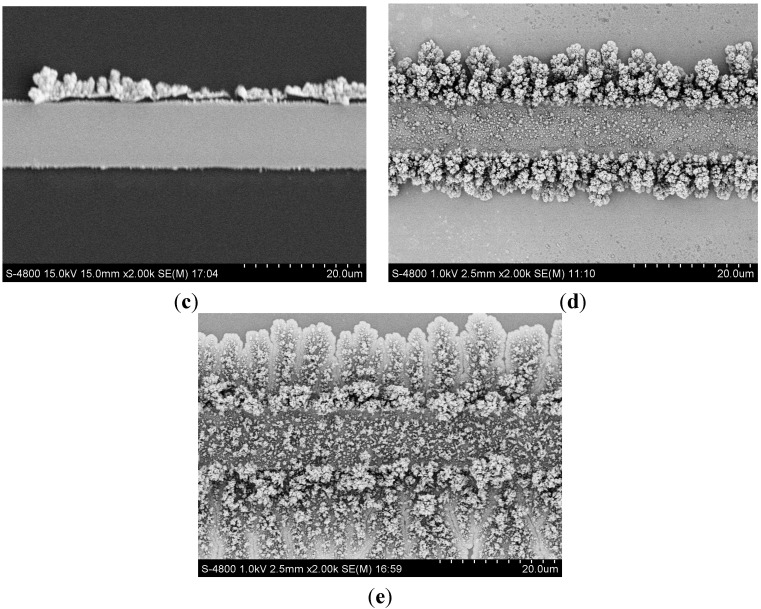
SEM images of palladium layer deposited under a fixed potential of (**a**) −0.3 V; (**b**) −0.4 V; (**c**) −0.5 V; (**d**) −0.56 V; (**e**) −0.62 V.

Different potentials were carried out to make out the suitable parameters for palladium electrodeposition. When a less negative deposition potential (−0.3 V~−0.5 V) was applied, gaps would be formed on the electrodeposited layer and some parts of the deposit got separated from the substrate (Figure 1a–c). We suppose that fewer crystal nuclei were formed at less negative potentials. Crystals grew and developed into slices with more deposits accumulated. The accumulation led to stress inside the deposit and then the deposit cracked on the boundaries of different slices and gaps were formed. The palladium layer was thus no longer adhesive to the substrate and sometimes got completely separated from the substrate. Accordingly, a more negative potential (−0.56 V) was applied and a well-deposited palladium layer (shown in Figure 1d) was observed. A more negative deposition potential resulted in dense nucleation. Crystallites were packed and stress distribution was balanced. In this case, the deposit kept adhesive to the substrate. The potential for deposition shouldn’t be too negative, as shown in Figure 1e. The palladium layer got so prosperous that it may lead to a high current output that the electrode cannot bear in subsequent modification steps.

#### 3.1.2. Electrodeposition of Tin

Tin was electrodeposited on the prepared palladium layer by cyclic voltammetry (CV). The high limit of CV was set to be −0.45 V *vs.* Ag/AgCl where charge transfer reduction of the metal ion approximately initiated. On the other hand, when the prepared palladium modified electrode was cycled in 20 mM SnCl_2_, the redox current increased significantly at more negative potentials. The increase was due to fierce hydrogen evolution enhanced by palladium. Fierce hydrogen evolution would damage the modification layer till a certain amount of tin was deposited and the palladium was covered. Hence, a relatively positive low limit of CV was applied to ensure milder hydrogen evolution. Consequently, the deposition potential of tin was applied from −0.45 V to −0.58 V.

#### 3.1.3. Characterization of Pd-Sn Electrode

**Figure 2:**
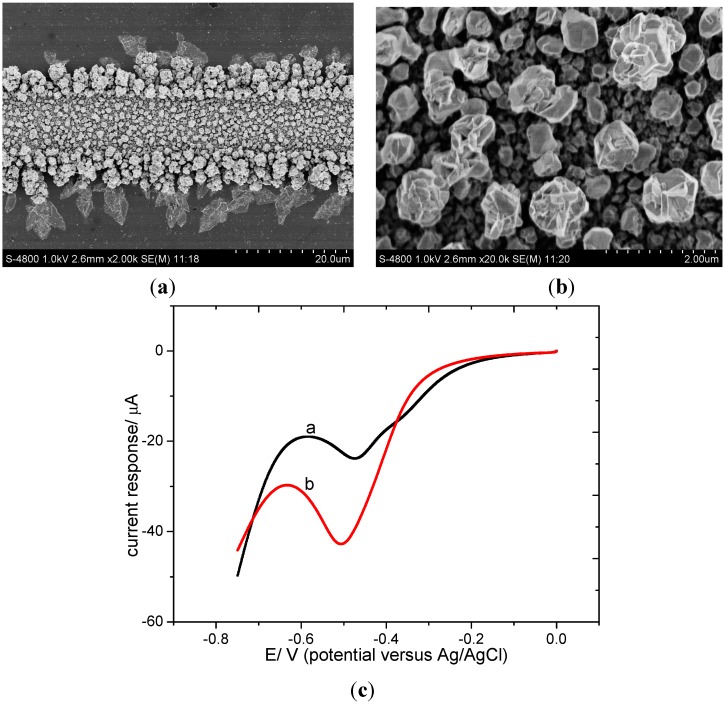
(**a**) SEM image of a single band of the Pd-Sn modified electrode (×2000); (**b**) SEM image of the surface of the Pd-Sn modified electrode (×20,000); (**c**) linear sweep voltammograms of Pd-Sn modified electrode testing in 0.01 M HClO_4_ respectively containing 0 mg/L (as N) KNO_3_ (curve a) and 5 mg/L (as N) KNO_3_ (curve b).

The SEM images of the surface morphology of the Pd-Sn modified microband electrode array are shown in Figure 2. Metal on the surface of the microband is piled up like gravel, forming a porous and rough structure. In general, the size of the metal gravel is about 200 nm. Such a kind of structure enlarges the electroactive area and enhances catalytic activity. In addition, in this way, a close contact of palladium and tin is acquired, which is beneficial for maintaining the catalytic activity [32]. EDS analysis results confirmed that the elemental composition of the deposition layer on the microband electrode array is palladium and tin. Linear sweep voltammograms (LSVs) of the electrode in nitrate solution (Figure 2c) show the basic behavior of the modified electrode. The response for nitrate at about −0.5 V and the inhibition of hydrogen evolution can also prove that tin and palladium coexist on the microband. The smaller peak in curve a is due to the reduction of dissolved oxygen and is regarded as the background.

### 3.2. Nitrate Determination

#### 3.2.1. Electrochemical Measurement with Different Modified Electrodes

**Figure 3:**
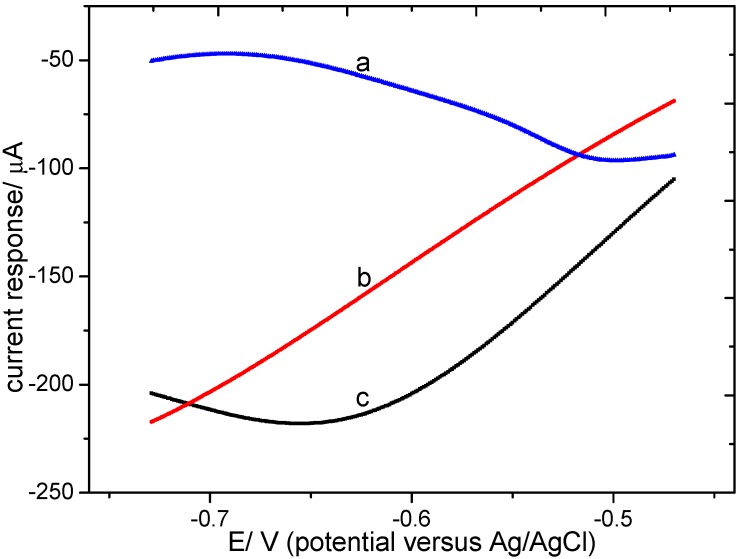
LSVs on (**a**) a palladium-tin modified electrode, (**b**) a bare electrode and (**c**) a palladium modified electrode in a 20 mg/L (as N) KNO_3_ acidic solution.

**Figure 4:**
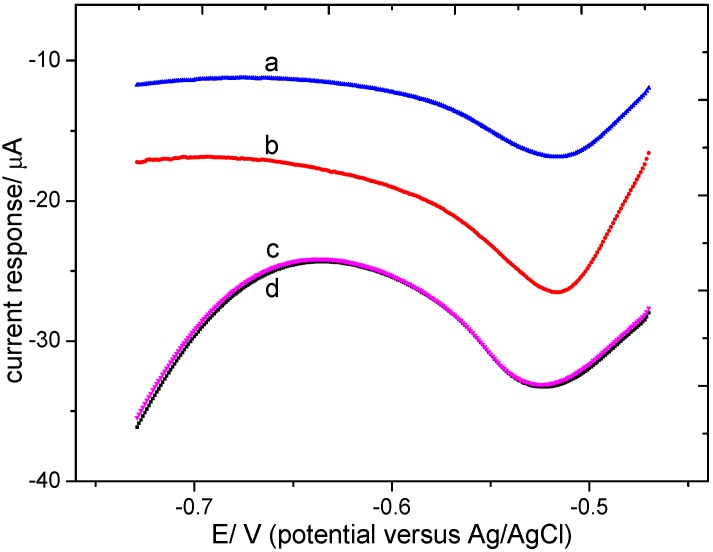
LSVs on (**a**) a tin modified electrode after several times of analysis, (**b**) a freshly deposited tin modified electrode, (**c**) a freshly deposited palladium-tin modified electrode and (**d**) a palladium-tin modified electrode after several times of analysis in a 3 mg/L (as N) KNO_3_ solution.

**Figure 5:**
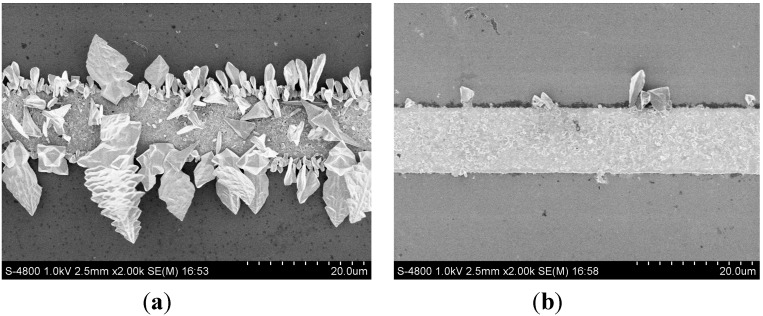
SEM images of (**a**) freshly deposited tin layer (**b**) tin layer after several times of LSV analysis.

The LSVs recorded for the bare microband electrode array, palladium modified electrode and the Pd-Sn modified electrode in 0.01 M HClO_4_ containing 20 mg/L (as N) KNO_3_ are displayed in Figure 3. The bare electrode exhibited high current owing to hydrogen evolution in the acidic medium (curve b). The palladium modified electrode showed a higher current response for hydrogen evolution for its better ability to absorb hydrogen than platinum. Only the Pd-Sn modified electrode showed clear current response for nitrate at about −0.5 V to confirm the catalytic activity towards nitrate reduction. LSVs recorded for the tin modified electrode and the Pd-Sn modified electrode in 0.01 M HClO_4_ containing 3 mg/L (as N) KNO_3_ are displayed in Figure 4. Tin modified electrode showed a sharp decline in current response in nitrate solution after about five times of LSV analysis (curve a), compared with a stronger current output examined in the same electrolyte when it’s freshly prepared. The tin modified electrode became invalid because the deposited layer was damaged, as was shown in Figure 5. The Pd-Sn modified electrode (curve c) showed clear current response and the response kept relatively stable after several times of LSV detection (curve d), which would be described in detail later when repeatability was examined. These results implied that palladium-tin coexistence has good catalytic activity of nitrate reduction and also enhances the short-term stability of the electrode.

#### 3.2.2. Optimization of the Electrolyte pH

**Figure 6:**
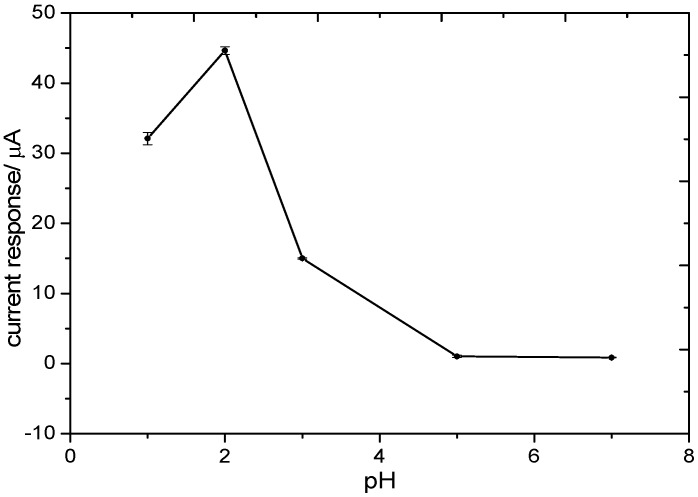
Current response by LSV at E = −0.51 V (potential versus Ag/AgCl) in 5 mg/L KNO_3_ (as N) and 0.01 M KClO_4_ solutions with different pH.

The supporting electrolyte was KClO_4_ and HClO_4_, which were stable in dilute solution. The detection was performed by linear sweep voltammetry (LSV) in 5 mg/L (as N) KNO_3_ solutions adjusted to different pH with HClO_4_. The potential was set from −0.47 V to −0.73 V, and the scan rate was 50 mV/s. The initial potential of LSV was set −0.47 V to avoid possible dissolution of tin on the electrode as it tends to oxidize under more positive potential of about −0.3 V to −0.4 V [33]. The relationship between the current response and pH is shown in Figure 6. The modified electrode exhibited a strong current response in acidic media while in neutral electrolyte the electrochemical reaction was comparatively negligible. The highest redox current appears at pH 2. In aqueous media, nitrate is firstly reduced to nitrite, which is the rate determining step and is followed by reduction to other substances including NO, N_2_, NH_3_, *etc.* The ratio of these reduction products depends strongly on many factors such as electrode material, electrolyte pH and additives in the solution [34]. As protons act as a reactant in the reduction steps, the oxidation ability of nitrate is thermodynamically better in acidic media than in neutral media [35]. The experiment result is consistent with the equation. Thus, the electrolyte with the pH value of 2 was chosen for further investigation and calibration.

#### 3.2.3. Nitrate Determination at the Pd-Sn Modified Electrode 

**Figure 7:**
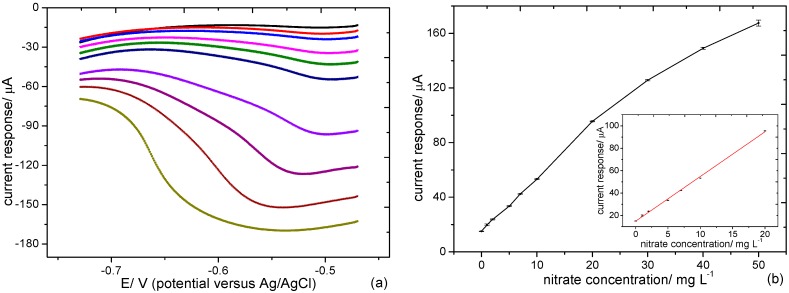
(**a**) Linear sweep voltammograms detailed the determination of nitrate. Voltammograms were recorded in 0, 1, 2, 5, 7, 10, 20, 30, 40, 50 mg/L (as N) KNO_3_ solutions (up to down); (**b**) The corresponding calibration plot showed current response when E = −0.51 V.

Simulated water samples containing supporting electrolyte and different concentrations of KNO_3_ were prepared. Figure 7a shows linear sweep voltammograms of a Pd-Sn modified microband electrode array in solutions containing different concentrations of nitrate ions. As can be seen, a large redox wave of the nitrate reduction species is observed at about −0.5 V *versus* Ag/AgCl reference, and the peak current increased with ascending nitrate concentration in the range of 1−50 mg/L. The inset of Figure 7b shows a calibration plot of peak current values measured in the voltammograms (E = –0.51 V) as a function of nitrate concentration in the range of 1~20 mg/L with the linear equation of y = 14.9 + 3.982x (R^2^ = 0.998). The limit of detection was estimated to be 0.19 mg/L (S/N = 3).

#### 3.2.4. Repeatability and Lifetime

The repeatability of the Pd-Sn modified electrode was examined by analyzing the current response of linear sweep voltammetry with the potential ranging from −0.47 V to −0.73 V in 3 mg/L KNO_3_ (pH = 2) for 12 times in succession. The relative standard deviation (RSD) of the peak current at the potential of −0.51 V for nitrate reduction was 2.35%, indicating a good repeatability of the electrode.

**Figure 8:**
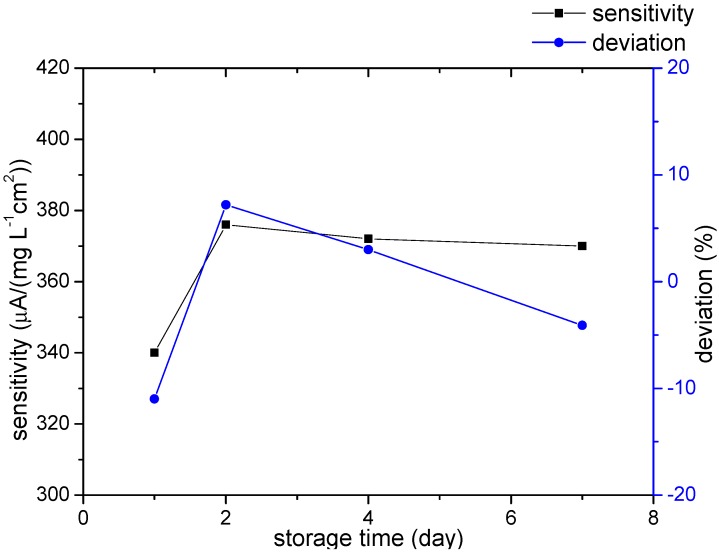
Analytical performance of the same electrode after different days-storage.

The modified electrode was calibrated each time before it was put into use. Three nitrate standard samples with nitrate concentrations of 1, 3, 7 mg/L (as N) were used for calibration. The simulated nitrate sample with a concentration of 5 mg/L (as N) was then detected by the modified electrode to see the deviation from the calibration. In this way, whether the electrode was still valid was verified and the lifetime could be estimated. The deviation at 5 mg/L nitrate from the calibration plot was below ±11.0% in the first seven days of repeated use and the sensitivity kept relatively stable between 340–376 μA/(mg·L^−1^·cm^2^), as shown in Figure 8. The results indicate that the analytical performance keeps stable and the electrode can be repeatedly used for at least a week. Similar experiments were carried out by other modified electrodes after they were stored for longer time. The electrode exhibited a deviation of −7.0% after 40 days, and a deviation of −6.6% after 60 days, showing that the modified electrode can still work properly after an at least 60-day-long storage. This can be attributed to the stable chemical property of Pd-Sn bimetallic composite.

**Table 1:** Comparison of analytical performance of some electrochemical nitrate sensors.

Catalyst	Working Electrode	Supporting Electrolyte	pH	Sensitivity (μA/(mg·L^−1^·cm^2^))	Lifetime	Literature
Palladium-tin	Platinum microband electrode array	0.01 M HClO_4_	2	398	More than 60 days	This paper
Palladium-tin	GC disk electrode (d = 3 mm)	0.05 M H_2_SO_4_	1	167	-	2006, [24]
Copper	Platinum microband electrode array	0.1 M Na_2_SO_4_	2	315	-	2015, [31]
Copper	Copper (d = 3 mm)	0.1 M Na_2_SO_4_	2	147	Renewable *in situ*	2007, [36]
Palladium-copper	Epoxy-copper electrode (d = 3 mm)	0.1 M PBS	7	17	-	2013, [37]
AgNPs	GC disk electrode (d = 3 mm)	0.1 M PBS	6.7	52	1 day	2013, [9]
PPy/Ag	Epoxy-glassy carbon rod	0.1 M Na_2_SO_4_	7	1.6	More than 60 days	2011, [38]
Macroporous Ag	ITO (0.6 cm × 1.2 cm)	1 M NaOH	14	9.29	-	2010, [8]

Table 1 shows the comparison of analytical performance for nitrate determination between the Pd-Sn modified electrode in this paper and other metal modified electrodes reported in literature. Some most commonly studied materials including copper and silver are listed in the comparison. As is indicated in Table 1, the Pd-Sn catalyst in our work exhibits good sensitivity among common metal catalysts listed below. Meanwhile, it shows a longer lifetime than other metal catalysts. A polymer catalyst with silver incorporated also shows good lifetime owing to the PPy matrix [38], but it exhibits much lower sensitivity than that in this work. In conclusion, the Pd-Sn catalyst has an advantage over other common catalysts for nitrate determination.

## 4. Conclusions

This work demonstrates a Pd-Sn modified microband electrode array used for nitrate determination. Pd-Sn bimetallic composite was electrodeposited, forming a gravel-like Pd-Sn layer. Preliminary detection was carried out to investigate its analytical performance. The electrode displayed good linearity and repeatability in low concentrations, indicating that Pd-Sn was a possible sensing material for nitrate determination in drinking water. Additional experiments were taken to verify that the modified electrode could be repeatedly used and confirmed that the coexistence of palladium and tin improves both the sensitivity and the lifetime of the nitrate sensor. Further investigations are still needed for optimization of sensitivity and lifetime. To summarize, the palladium-tin modified microband electrode array has the potential to be a nitrate sensor with high sensitivity and long lifetime.
